# Systematic review of Doppler for detecting intrapartum fetal heart abnormalities and measuring perinatal mortality in low‐ and middle‐income countries

**DOI:** 10.1002/ijgo.13014

**Published:** 2019-12-05

**Authors:** Marya Plotkin, Benjamin Kamala, Jim Ricca, Linda Fogarty, Sheena Currie, Hussein Kidanto, Stephanie B. Wheeler

**Affiliations:** ^1^ USAID's Maternal and Child Survival Program/Jhpiego Washington DC USA; ^2^ Faculty of Health Sciences University of Stavanger Stavanger Norway; ^3^ Jhpiego, Baltimore MD USA; ^4^ School of Medicine Aga Khan University Dar es Salaam Tanzania; ^5^ Department of Health Policy and Management Gillings School of Global Public Health University of North Carolina at Chapel Hill Chapel Hill NC USA

**Keywords:** Doppler, Fetal heart monitoring, Intrapartum, Low‐ and middle income countries (LMIC), Pinard stethoscope

## Abstract

**Background:**

Using Doppler to improve detection of intrapartum fetal heart rate (FHR) abnormalities coupled with appropriate, timely intrapartum care in low‐ and middle‐income countries (LMIC) can save lives.

**Objective:**

To review studies using Doppler to improve detection of intrapartum FHR abnormalities and intrapartum care quality in LMIC health facilities.

**Search strategy:**

PubMed, Web of Science, Embase, Global Health, and Scopus were searched from inception to October 2018 by combining terms for Doppler, perinatal outcomes, and FHR monitoring.

**Selection criteria:**

Selected studies compared Doppler and Pinard stethoscope for detecting/monitoring intrapartum FHR, or described provider and maternal preferences for FHR monitoring in LMIC settings.

**Data collection and analysis:**

Two team members independently screened and collected data. Risk of bias was assessed by Cochrane EPOC criteria.

**Results:**

Eleven studies from eight countries were included. Doppler was superior at detecting abnormal intrapartum FHR as compared with Pinard stethoscope, but was not associated with improved perinatal outcomes. Using Doppler on admission helped to accurately measure perinatal deaths occurring after facility admission.

**Conclusion:**

Studies and program learning are needed to translate improved detection of FHR abnormalities to improved case management in LMICs. Doppler should be used to calculate a facility indicator of intrapartum care quality.

**PROSPERO registration:** CRD42019121924.

## Abstract 2

Doppler is superior to Pinard stethoscope for detecting fetal heart rate abnormalities. Without a focus on prompt clinical management, improving perinatal outcomes will be difficult.

## 1 INTRODUCTION

Worldwide, an estimated 2 million early neonatal deaths occur in low‐ to middle‐income countries (LMIC) annually, including 904 000 intrapartum‐related neonatal deaths and 1.02 million fresh stillbirths.^1^, ^2^ Nearly all intrapartum stillbirths and neonatal deaths that occur in health facilities can be prevented by good obstetric care,^3^ essential newborn care, and prompt identification and resuscitation of asphyxiated neonates.^4^

Interruption of placental blood flow during labor can result in fetal heart rate (FHR) acceleration, deceleration, bradycardia (<120 beats per minute) and/or tachycardia (>160 bpm). Such FHR abnormalities have been associated with low Apgar score, intrapartum stillbirth, and neonatal death.^5^, ^6^ Early detection of FHR abnormalities, linked to timely and appropriate obstetric case management practices, can potentially reduce adverse perinatal outcomes.

A 2017 Cochrane review found that continuous monitoring of FHR by using cardiotocography—the standard of care in high‐income countries—was associated with increased numbers of cesarean and assisted deliveries, without a corresponding decrease in adverse newborn outcomes.^7^ This may have contributed to the WHO's recommendation to use intermittent FHR monitoring.^8^, ^9^ That guidance, however, contains no recommendation of which device (Pinard stethoscope or Doppler) should be used for auscultation^9^; as a result, many studies have examined the effectiveness of Doppler for intrapartum FHR monitoring in LMIC settings.

The utility of Doppler in the intrapartum care setting is not limited to the diagnosis of fetal heart abnormalities. The importance of an indicator that can be used to track intrapartum deaths in health facilities was noted in a call to action in the *Lancet* in 2007.^10^ Subsequent studies have used Doppler to confirm timing of fetal demise in order to measure stillbirths and neonatal deaths that occur after admission to the health facility.

Maternal preference may increasingly influence which method is used for FHR monitoring in LMIC settings.^9^ Some laboring women have noted that hearing the fetal heartbeat amplified by Doppler is a positive experience, and others have reported that the Pinard fetoscope causes discomfort.^11^, ^12^ To our knowledge, maternal preferences for the method of FHR monitoring in the LMIC health facility setting have not been systematically described.

The aim of the present systematic review was, therefore, to determine (1) whether Doppler for intrapartum FHR monitoring is associated with a decrease in adverse perinatal outcomes; (2) whether Doppler can be effectively used to calculate a facility‐based indicator of perinatal mortality; and (3) whether women and healthcare providers express a preference for Doppler over Pinard stethoscope for intrapartum FHR monitoring in LMIC settings.

## 2 MATERIALS AND METHODS

### 2.1 Search strategy and search terms

The review was registered with PROSPERO (reference CRD42019121924) and followed guidelines detailed in the PRISMA (Preferred Reporting Items for Systematic Reviews and Meta‐Analyses) statement.^13^ The following databases were searched from inception up until October 31, 2018: PubMed, Web of Science, Embase, Global Health, and Scopus.

The following search terms were used: (Doppler OR fetoscope OR Pinard) AND (newborn OR labor OR labour OR delivery OR perinatal OR intrapartum OR stillbirth OR still birth OR fetal OR foetal OR fetus OR neonatal OR “intermittent fetal heart rate monitoring” OR “fetal heart”). Searches were limited to English and had no date restriction. Both American and UK English spelling was considered in the search terms.

Records retrieved through the searches were imported into Covidence systematic review software (Veritas Health Innovation, Melbourne, Australia) and duplicates were removed automatically. Additional studies were identified by using backward searches (snowballing) of references in relevant articles.

### 2.2 Inclusion criteria

For inclusion, the studies must have been conducted in a LMIC, assessed an intervention that included Doppler in the intrapartum (not pregnancy) period, have been conducted in a health facility or with health facility staff, have tested use of Doppler to improve the detection of FHR abnormalities to inform intrapartum interventions, address maternal or healthcare provider preference for tools of FHR monitoring during the intrapartum period, or have tested the validity or application of an indicator in which Doppler is used to assess timing of fetal demise. Systematic reviews, case reports, abstracts, and unpublished reports were excluded.

### 2.3 Data collection and analysis

Titles and abstracts were screened on the basis of the inclusion and exclusion criteria. At this stage, the abstract was perused to assess fit to the given criteria. Studies were selected for inclusion by two researchers (MP, BK), working independently. Disagreements between the two authors were resolved by discussion and review by a third researcher (SW).

After screening, full text versions of eligible studies were examined. Data were extracted by using a pre‐defined data extraction form. Abstracted data included study setting and design, study outcome measures, key findings, summary of limitations, type and characteristics of the intervention, outcome measures, and effect of the intervention on the outcome measures. Qualitative data were described by using textual narrative synthesis, as recommended for systematic reviews. Risk of bias and quality of evidence were assessed by using the Cochrane Effective Practice and Organisation of Care (EPOC) criteria.^14^

## 3 RESULTS

### 3.1 Search results and included studies

**Figure 1:**
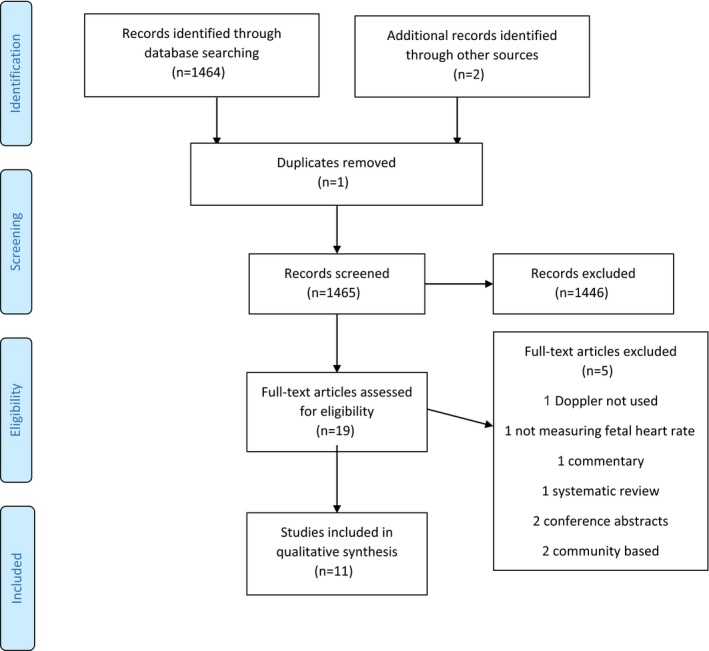
PRISMA flow diagram depicting systematic search strategy.

The initial search yielded 1464 records. After de‐duplication, 1463 articles remained. Of these, 1446 articles did not meet the inclusion criteria and the remaining 19 studies were reviewed in full. Of these, 11 studies from Tanzania, Uganda, South Africa, India, Pakistan, Democratic Republic of Congo, Kenya, and Zimbabwe met the inclusion criteria and were included in the review (Fig. 1).

Of the 11 included studies, all but one^15^ were published in the past 10 years. Six studies assessed the effectiveness of Doppler to detect abnormal FHR during intrapartum care, two studies assessed Doppler‐based verification of FHR on admission for calculation of an indicator of perinatal mortality, and three studies assessed maternal or healthcare provider preferences for method of intrapartum FHR monitoring.

### 3.2 FHR abnormalities and adverse perinatal outcomes

Six studies addressed the effectiveness of Doppler versus Pinard stethoscope for the detection of abnormal FHR during intermittent or continuous FHR monitoring in the intrapartum period (Table 1).^15^, ^16^, ^17^, ^18^, ^19^, ^20^ All six studies had secondary outcome measures of adverse perinatal outcomes. Two compared continuous fetal monitoring using a Doppler with intermittent monitoring using the Pinard stethoscope. Types of Doppler used in the studies included the PowerFree Education Technology Wind‐up Fetal Doppler,^16^ Freeplay (wind‐up) Doppler,^17^ Moyo strap‐on Doppler using the continuous or intermittent monitoring function,^18^, ^19^ and the Huntleigh pocket Doppler.^15^

**Table 1:** Studies on the effectiveness of FHR monitoring by Doppler to reduce perinatal mortality

Ref. (year)	Country	Study objective	Study design	Study population	Clinical management differences	Perinatal outcome or abnormal FHR detection
16 (2017)	Uganda	To compare intermittent fetal heart monitoring between Doppler and Pinard for detection of FHR abnormalities (primary outcome), and intrapartum stillbirth and death within first 24 h of life (secondary outcomes)	Two‐arm RCT	n=1987 women at one peri‐urban hospital Doppler, n=1000 Pinard, n=987	No differences in rate of cesarean deliveries	Higher detection of FHR abnormalities in the Doppler arm (incidence rate ratio, 1.61; 95% CI, 1.13–2.30; *P*=0.008). No difference in rate of intrapartum stillbirth, neonatal death, Apgar score <7 at 5 min, or admission to NICU
18 (2018)	Tanzania	To compare intermittent fetal heart monitoring between Doppler and Pinard for detection of FHR abnormalities (primary outcome), and intrapartum stillbirth, neonatal death, time to delivery, and mode of delivery (secondary outcomes)	Two‐arm RCT	2844 women at Tanzania's national referral hospital Doppler, n=1421 Pinard, n=1423	No difference in time between detection of an abnormal FHR to delivery	Higher detection of FHR abnormalities in Doppler (6.0%) vs Pinard (3.9%) arm (aOR, 1.59; *P*=0.008). Overall, no difference in perinatal death. Among newborns with abnormal FHR delivered vaginally, fewer adverse outcomes in Doppler (16.3%) than in Pinard (43.5%) arm (*P*=0.021). No difference in Apgar score <7, bag–mask ventilation, mode of delivery, perinatal admission to NICU, or perinatal deaths
15 (1994)	Zimbabwe	To compare effectiveness of CTG, intermittent monitoring with Doppler, intermittent monitoring with Pinard by a research midwife, and intermittent monitoring with a Pinard by facility midwife on detection of abnormal FHR (primary outcome) and cesarean delivery, neonatal mortality, and admission to NICU (secondary outcomes)	Four‐arm RCT Doppler for intermittent monitoring, CTG, Pinard by research midwife (gold standard), Pinard by facility midwife (routine monitoring)	n=1255 women at one urban referral hospital Doppler, n=312 Pinard by research midwife, n=310 Pinard by facility midwife, n=315 CTG, n=318	No difference in time between detection of FHR abnormality and delivery among the 4 groups. Cesarean more common in CTG (28%) and Doppler (24%) arms than in Pinard arms with research (10%) and facility (15%) midwives. Fetal distress was indication for cesarean in 63% of CTG and 67% of Doppler arms, each significantly higher than Pinard arms (41%)	Compared with routine monitoring, RR of detecting abnormal FHR was 6.1 (95% CI, 4.2–8.8) with CTG, 3.6 (95% CI, 2.4–5.3) with Doppler, and 1.7 (95% CI, 1.1–2.7) with the Pinard/research midwife. Stillbirth or neonatal death was 3% (CTG); 0.6% (Doppler); 2% (Pinard with research midwife) and 3% (routine monitoring). Significantly fewer neonates were admitted to NICU in the Doppler vs other arms
17 (2018)	Tanzania	To compare intermittent fetal heart monitoring between Doppler and Pinard for detection of FHR abnormalities (primary outcome) and intrapartum stillbirth, neonatal death and admission to NICU within 24 h (secondary outcomes)	Two‐arm RCT	n=2684 women at one rural referral hospital Doppler, n=1309 Pinard, n=1375	No difference in time between detection of abnormal FHR to delivery. No difference in cesarean delivery rates	Abnormal FHR detected in 4.2% of Doppler vs 3.1% of Pinard arm, not significant (RR, 1.38; 95% CI, 0.93–2.04). No difference in adverse perinatal outcomes or bag–mask ventilation between Pinard and Doppler arms
19 (2018)	Tanzania	To assess the effect of introducing continuous FHR monitoring on detection of abnormal FHR (primary outcome); and time to delivery, time from detection of abnormal FHR to delivery, and intrauterine resuscitation (secondary outcomes)	Observational pre‐ and post‐intervention	At one urban referral hospital, n=1640 women enrolled at the pre‐implementation stage and n=2442 at the implementation stage	Higher rate of cesarean observed post‐intervention (5.4%) vs pre‐intervention (2.6%) (*P*<0.001); Cause of cesarean was fetal distress in 48% of cases post‐intervention vs 35% pre‐intervention. Median time from last FHR assessment to delivery was 60 min pre‐intervention vs 45 min post‐intervention (*P*<0.001)	Continuous FHR monitoring with Doppler (post‐intervention) was associated with 6.9‐fold increased detection of abnormal FHR vs routine FHR monitoring with Pinard (pre‐intervention)
20 (2018)	Tanzania	To compare continuous fetal heart monitoring by Doppler and intermittent monitoring by Pinard for detection of FHR abnormalities (primary outcome) and intrapartum stillbirth, neonatal death, mode of delivery, 5‐min Apgar score, bag–mask ventilation, time from abnormal FHR detection to delivery, adverse fresh stillbirth, neonatal death within 24 h, and admission to NICU (secondary outcomes)	Two‐arm RCT	n=2652 women at one rural referral hospital Doppler with continuous monitoring, n=1340 Doppler with intermittent monitoring, n=1312	Increased rate of intrauterine resuscitations in continuous vs intermittent monitoring groups (6.6% vs 3.2%; RR 2.07, 95% CI 1.4–2.9; *P*<0.001). Fetal heart distress was the cause of 20.2% of cesareans in continuous vs 7.4% in intermittent groups (2.79; 95% CI, 1.7–4.6, *P*<0.001). Median time interval between detection of abnormal FHR to delivery was shorter in continuous (52 min) than in intermittent 75 min) group (*P*<0.04)	Continuous FHR monitoring with Doppler detected abnormal FHR in 8.1% vs 3.0% of women in intermittent monitoring group (RR 2.64, 95% CI 1.8–3.7; *P*<0.001). No significant differences in adverse outcomes between groups

Abbreviations: aOR, adjusted odds ratio; CI, confidence interval; CTG, cardiotocography; FHR, fetal heart rate; NICU, neonatal intensive care unit; RCT, randomized controlled trial; RR, risk ratio.

#### 3.2.1 Findings on detection of abnormal FHR

All but one study^17^ showed that Doppler significantly increased the detection of abnormal FHR relative to Pinard (Table 1), whether with continuous monitoring (adjusted odds ratio [AOR], 6.90; 95% confidence interval [CI], 3.89–12.24)^19^; risk ratio [RR], 2.64; 95% CI, 1.8–3.7^20^) or with intermittent monitoring (incidence rate ratio, 1.61; 95% CI, 1.13–2.30^16^; AOR, 1.59; 95% CI, 1.13–2.26; *P*=0.008^18^; RR, 3.6; 95% CI, 2.4–5.3^15^). The study that showed no difference in detection of abnormal FHR reported that this was likely to be due to a type 2 error.^17^

#### 3.2.2 Findings on adverse perinatal outcomes

Adverse perinatal outcomes were defined as intrapartum stillbirth, neonatal death within 24 hours, neonatal seizures, hypoxic ischemic encephalopathy, bag and mask ventilation, or admission to the neonatal intensive care unit (NICU). Two studies documented a reduction in perinatal adverse events associated with intermittent Doppler monitoring of intrapartum FHR as compared with intermittent monitoring with the Pinard fetoscope.^15^, ^18^ In the oldest study, Mahomed et al.^15^ reported a reduction of perinatal mortality in the arm using Doppler for intermittent monitoring, with neonatal death rates of 0.6% in the Doppler arm as compared with 2%–3% in the two Pinard arms. No statistical data were presented to demonstrate the significance of the finding.

In a more recent study in Tanzania, among newborns with abnormal intrapartum FHR who were delivered vaginally, lower rates of adverse outcomes (composite of fresh stillbirth, perinatal death, and NICU admission) were seen in the Doppler than in the Pinard arm (16.3% vs 45.3%, *P*=0.021).^18^ In the same study, however, there was no decline in adverse perinatal outcomes when all newborns in the study were considered. In the other four studies, no difference in adverse perinatal outcomes was seen between Doppler and Pinard fetoscope for FHR monitoring (Table 1).^15^, ^16^, ^17^, ^19^

#### 3.2.3 Findings on clinical management associated with abnormal FHR

Multiple studies looked at intrapartum clinical management procedures that would be expected to increase after detection of abnormal FHR and might be associated with a reduction in perinatal mortality. These measures included cesarean delivery,^15^, ^16^, ^17^, ^18^, ^20^ shortening the length of time from abnormal FHR detection to delivery,^15^, ^16^, ^17^, ^18^, ^20^ vacuum delivery, NICU admission, and intrauterine resuscitation.^20^

Two studies showed a higher rate of cesarean delivery with use of Doppler. In a randomized controlled trial (RCT) in Zimbabwe, the relative risk of cesarean after Doppler monitoring as compared with routine monitoring with Pinard was 1.6 (95% CI, 1.2–2.0).^15^ In an observational study in Tanzania, cesarean rates were 5.4% for women with continuous Doppler monitoring, as compared with 2.6% for those with intermittent Pinard monitoring (*P*<0.001).^19^ Other studies in Uganda^16^ and Tanzania^17^, ^18^ showed no difference in cesarean rates between Doppler and Pinard groups.

In another RCT in Tanzania, an increase in risk of intrauterine resuscitation was observed for women continuously monitored with Doppler as compared with those intermittently monitored with Pinard (RR, 2.07; 95% CI, 1.4–2.9); as described above, there was no difference in adverse perinatal outcomes between the two arms.^20^

In Tanzania, two RCTs of intermittent monitoring with Doppler versus intermittent monitoring with Pinard did not find a difference in time from abnormal FHR detection to delivery between the two arms.^17^, ^18^ In Zimbabwe, there was no difference in mean duration of labor among the four study groups.^15^ The observational study in Tanzania found that continuous FHR monitoring with Doppler was associated with a shorter time from last FHR assessment to delivery (median 45 minutes post‐ vs 60 minutes pre‐intervention, *P*<0.001).^19^ The RCT in Uganda did not report any measure of time associated with clinical management of the women.^16^

#### 3.2.4 Risk of bias and quality of evidence

For the six studies, risk of bias and quality of evidence were assessed by Cochrane EPOC criteria.^14^ The most pervasive risk in all of the RCTs was the lack of blinding regarding the device that the participants and study staff used (Table 2). Generation of the randomization sequence was unclear or undescribed in all studies except for an RCT at Muhimbili Hospital in Tanzania, where a computer‐generated sequence was created by an independent researcher.^18^ All studies had low risk of incomplete outcome data reporting and were free of selective reporting (all stated outcomes were reported).

**Table 2:** Risk of bias and strength of evidence using Cochrane criteria for assessment of bias in EPOC studies

Study/risk of bias	Random sequence generation	Allocation concealment	Blinding of women and personnel	Incomplete outcome data	Free of selective reporting	Blinding of outcome assessment	Baseline outcomes similar	Free of contamination	Baseline similar variables
15									
Judgement	Unclear risk	Low risk	High risk	Low risk	Low risk	High risk	Low risk	Low risk	Low risk
Description	Randomized sequence generation not described	Sequentially numbered opaque sealed envelopes	Blinding of both clinicians and women not possible	Proportion of missing data unlikely to change the study result	All outcomes reported	Outcomes assessors were not blind	No outcomes at beginning of study	All arms received allocated interventions; no crossing over	No important differences in study groups (*P*<0.05)
16									
Judgement	Unclear risk	Low risk	High risk	Low risk	Low risk	High risk	Low risk	Low risk	Low risk
Description	Randomized sequence generation not described	Sequentially numbered opaque sealed envelopes	Blinding of both clinicians and women not possible	Proportion of missing data unlikely to change the study result	All outcomes reported	Outcome assessors were not blind	No outcomes at beginning of study	All arms received allocated interventions; no crossing over	Baseline variables similar (*P*<0.05)
17									
Judgement	Unclear risk	Low risk	High risk	Low risk	Low risk	High risk	Low risk	Low risk	Low risk
Description	Randomized sequence generation not described	Sequentially numbered opaque sealed envelopes	Blinding of both clinicians and women not possible	Completed follow‐up per protocol	All primary and secondary outcomes reported	Not possible to blind outcomes for assessors	No outcomes at beginning of study	Allocated interventions adhered to	Baseline variables were (*P*<0.05)
20									
Judgement	Unclear risk	Low risk	High risk	Low risk	Low risk	High risk	Low risk	Low risk	Low risk
Description	Randomized sequence generation not described	Sequentially numbered opaque sealed envelopes	Blinding of both clinicians and women not possible	Completed follow‐up per protocol	All primary and secondary outcomes reported	Not possible to blind outcomes for assessors	No outcomes at beginning of study	Allocated interventions adhered to	Baseline variables similar (*P*<0.05)
19									
Judgement	Unclear risk	Low risk	High risk	Low risk	Low risk	High risk	Low risk	Low risk	Low risk
Description	No randomization; controlled before–after study	Controlled before‐after studies	Blinding of both clinicians and women not possible	Completed follow‐up per protocol	All primary and secondary outcomes reported	Not possible to blind outcomes for assessors	All mothers had normal FHR on admission	Unlikely as the two interventions took place at different times	Imbalances adjusted in the regression models
18									
Judgement	Low risk	Low risk	High risk	Low risk	Low risk	High risk	Low risk	Low risk	Low risk
Description	Randomizedsequence computer‐generated by independent statistician	Sequentially numbered opaque sealed envelopes	Blinding of both clinicians and women not possible	Completed follow‐up per protocol	All primary and secondary outcomes reported	Not possible to blind outcomes for assessors	All mothers had normal FHR on admission	Allocated interventions adhered to	Adjusted for baseline imbalances by logistic regression, multinomial regression, and linear regression
23									
Judgement	High risk	High risk	High risk	Low risk	Low risk	High risk	Unclear risk	Unclear risk	Unclear risk
Description	No randomization; cross‐sectional study	No randomization; cross‐sectional study	No blinding; cross‐sectional study	All data obtained	All outcomes reported	No blinding because of study design	Not described	Not described	Not described

Abbreviations: EPOC, effective practice and organization of care.

All studies were deemed to have low risk of contamination because the arms adhered to allocated interventions. Lastly, four studies demonstrated no significant baseline differences among the study groups and thus had low risk of bias associated with different baseline characteristics; the other two studies adjusted for baseline characteristics in the analysis.^18^, ^19^

### 3.3 Doppler as a tool for improving measurement of facility perinatal death

Two studies assessed the feasibility and validity of measurements of perinatal mortality in health facilities based on using Doppler to verify the presence or absence of an FHR on admission to labor and delivery services^21^, ^22^ (Table 3). A multi‐country study was conducted to determine the level of potentially preventable perinatal deaths occurring in study facilities and to describe the feasibility of the measure.^21^ It found that 40%–45% of intrapartum deaths occurring in‐facility were potentially preventable (based on the presence of positive fetal heart sounds on admission) and deemed that measurement of the Doppler‐based indicator would be feasible.

**Table 3:** Studies on the measurement of health facility‐occurring perinatal mortality using Doppler

Study (year)	Country	Study objective	Study design	Indicator	Definition	Data source for indicator	Population size/sample size	Key findings
21 (2013)	Pakistan, India, DRC, Kenya	To quantify the proportion of perinatal deaths that occurred in the facility setting and were potentially preventable, as a demonstration for potential scale‐up to assess quality of intrapartum care	Prospective study in which FHR was assessed by Doppler and basic information was recorded from women admitted to labor	Perinatal mortality rate per 1000 deliveries	Stillbirth and neonatal death before discharge per 1000 deliveries (stratified by occurrence in hospital, or neonate <2500 g at birth)	Personal medical record	n=3555 women and n=3593 neonates in 6 hospitals in 5 countries	Approximately 40% of perinatal mortality occurred in‐hospital, and was potentially preventable with better care. Perinatal mortality rate was 34 deaths per 1000 deliveries overall, and 13 per 1000 deliveries in‐facility. Restricted to neonates weighing 2500 g or more, perinatal mortality rate was 22 per 1000 deliveries overall, and 9.4 per 1000 deliveries in‐facility
22 (2018)	Tanzania	To validate an indicator of facility perinatal mortality by (1) comparing perinatal outcomes (macerated stillbirth, fresh stillbirth, neonatal death) as recorded in the facility register to gold‐standard audits, and (2) calculating an indicator for the study sites	A validation study in which audits were conducted on 128 perinatal deaths recorded in the health facility's national health information system register over 6 mo	Facility perinatal mortality indicator	Fresh stillbirth and very early neonatal deaths divided by all women admitted to the facility with a FHR detected	National health information system maternity register	n=9687 women admitted to labor and delivery services in 10 health facilities in the Kagera region of Tanzania; n=d128 perinatal deaths were audited to assess validity of register‐recorded classification	Sensitivity and specificity of register outcomes to predict audit outcomes ranged from 95.7% to 100%, validating the accuracy of register data for calculating the indicator. Rates of perinatal mortality occurring in‐facility ranged from 4.2% (regional hospital); 1.5%–2.7% (district hospitals); and 0.3%–0.5% (health centers); Use of Doppler on admission and recording the FHR in the register produced a more specific measure as compared with crude perinatal death rate, which included macerated stillbirth and was thus less reflective of quality of intrapartum care

Abbreviations: DRC, Democratic Republic of Congo; FHR, fetal heart rate.

In a study in Tanzania, healthcare providers used Doppler to check FHR on admission to the facility and recorded the findings in the national facility register.^22^ Perinatal deaths recorded in the register during the study period were verified through use of perinatal death audit. The aim of the study was to create an indicator of facility perinatal mortality that can be tracked through the national health information system. The study authors recommended that the indicator should be used to track perinatal deaths occurring after admission to the facility and that the results of indicator tracking should be linked to quality improvement initiatives.

### 3.4 Healthcare provider and maternal preferences for Doppler versus Pinard stethoscope

Three studies examined maternal or healthcare provider preferences for Pinard fetoscope as compared with Doppler for intrapartum FHR monitoring^11^, ^23^, ^24^ (Table 4). In a South African study that compared maternal preferences for Doppler, Pinard, and cardiotocography, 74% of women reported Doppler as their first choice.^23^

**Table 4:** Studies related to healthcare provider or maternal preference for Doppler vs Pinard

Study (year)	Country	Primary research question	Study design	Population	Data source	Key findings	Recommendations
11 (2018)	Tanzania	To describe attitudes and perceptions of women in labor continuously monitored with a strap‐on Doppler device and their perceptions of quality of intrapartum care	A cross‐sectional qualitative study; in‐depth interviews conducted within 12–24 h of delivery among women who delivered in an urban hospital	Multiparous women monitored using a continuous Doppler monitoring system during their delivery; only women with positive birth outcomes were interviewed	n=20 interviews	Use of the monitor positively affected the women's birth experience by providing reassurance about the wellbeing of the fetus. Women believed that use of the device improved care provided by the health facility staff through increased communication and attention from birth attendants; Participants were given little to no information about the purpose or functions of the device, and thus did not fully understand and often overestimated its capabilities	On the introduction of Doppler for intrapartum care, information should be included in counseling during prenatal care and/or in the early stages of labor. Information should include limitations of the device to avoid overestimation of its capabilities
24 (2018)	Tanzania	To explore midwives’ perceptions on using either Doppler, Pinard fetoscope, or Freeplay wind‐up for intermittent FHR monitoring in a rural hospital	Cross‐sectional qualitative assessment using FGDs	Midwives employed at the study hospital for at least 6 mo; trained in use of both Doppler and Pinard fetoscope	n=5 FGDs held with 25 participants	The study did not reveal a common and clear preference for Doppler vs fetoscope for FHR assessment. Three themes emerged: (1) sufficient training and experience in using a device, (2) perceived ability of devices to produce reliable measurements, and (3) convenience of use and comfort of the device	Regular training to make use of Doppler easier, and equal availability of fetoscopes and Doppler in labor wards. More research is needed to address practitioners’ preferences on best ways to conduct FHR monitoring
23 (2009)	South Africa	To document preferences on 3 methods of FHR assessment (Doppler, Pinard fetoscope, and cardiotocography) by laboring women	Cross‐sectional study based on interview with women in labor	Women in the first stage of labor. In the course of 30 min, women were assessed with wind‐up Doppler, Pinard fetoscope and cardiotocography in succession. Women were then asked to rank their first and second choice	n=97 women were interviewed	72 of 97 women preferred Doppler for assessing FHR in the first stage of labor	FHR monitoring by Doppler was found to be more acceptable to laboring women as compared with Pinard stethoscope or cardiotocography

Abbreviations: FGD, focus group discussion; FHR, fetal heart rate.

In a qualitative assessment of women who were continuously monitored with a strap‐on Doppler device in Tanzania, women were reassured by the sound of the heartbeat and felt that the Doppler made healthcare providers more attentive.^11^ The authors concluded that, although using Doppler for intrapartum FHR monitoring was appreciated by the laboring women, further use of this device should be accompanied by educating women on its capabilities.

In a Tanzanian RCT among nurses and nurse‐midwives who had used either Doppler or Pinard fetoscope for intermittent FHR monitoring, the nurses and midwives tended to prefer the device with which they were most familiar.^24^ The study's recommendation was to include adequate education on Doppler for healthcare providers when introducing the device into pre‐service and/or professional training.

All three studies had notable limitations that lessened the generalizability of results. The Tanzanian RCT was conducted with relatively few midwives from one health facility, and reflected device use based on random assignment rather than on provider preference.^11^ The South African study, which compared maternal preferences among Doppler, Pinard and cardiotocography, did not test FHR monitoring throughout labor, but rather at a single point during the first stage of labor.^23^ In addition, the authors did not address the potential effects of being in active labor while giving feedback, nor did they describe the information that they provided to participants about the efficacy of the devices for FHR monitoring. Lastly, the study did not provide statistics to test significance of the findings.

The qualitative study from Tanzania, which assessed women's perceptions on Doppler for continuous monitoring of FHR during labor, reflected views from women who attended services at one facility and included only women who had healthy newborns.^11^ Interviews were conducted before discharge from the facility, which might have affected the women's openness to answer questions honestly.

## 4 DISCUSSION

An estimated 1 million neonatal deaths and half of all maternal deaths might be prevented with higher quality maternal and newborn care.^25^ Lack of intrapartum monitoring of FHR according to standards contributes to persistently high levels of perinatal and neonatal death in LMIC.^2^, ^26^ Although assessment of the fetus at the time of admission to labor and delivery services is supposed to be routine,^27^ in practice, there is evidence to suggest that FHR is often not assessed 17 and/or not recorded 21 in LMIC health facilities.

A study of perinatal death audits in Tanzania showed that poor FHR monitoring was associated with more than 40% of the deaths.^27^ In Zanzibar, poor quality of intrapartum care was a determinant in almost all stillbirths that occurred in the hospital, with median time from last fetal heart assessment to fetal death or delivery being 210 minutes.^28^ These persistent gaps in quality of intrapartum FHR monitoring have consequences for the survival of neonates, and new means to close them are needed. To this end, the present study has reviewed the ways in which Doppler has been used in intrapartum care in LMIC health facilities: namely, to improve the detection of intrapartum FHR abnormalities, to respond to maternal and provider preferences, and to improve measurements of quality of intrapartum care.

### 4.1 Doppler and perinatal mortality

Except in one instance,^19^ none of the reviewed studies reported a reduction of perinatal mortality associated with use of Doppler for FHR monitoring as compared with Pinard fetoscope. This finding echoes that of a broader systematic review of intrapartum fetal surveillance in LMIC.^29^ In multiple studies where Doppler was used for FHR monitoring,^15^, ^16^, ^17^, ^18^, ^20^ although the detection of abnormal FHR increased, proxy measures of clinical management following this event (cesarean delivery, shortened time to delivery) did not increase. The implication of this finding is that introduction of Doppler to improve early detection of intrapartum FHR abnormalities needs stronger support for the stages that follow detection of the abnormality. This may include job aids, such as the decision trees developed by the UK National Institute for Health and Care Excellence,^30^ protocols addressing case management or referral processes, or other structural support to improve the quality of intrapartum care after detection of abnormal FHR.

Continuous monitoring of FHR has been associated with an increase in cesarean delivery, which may not benefit the mother.^8^ Given WHO guidance cautioning about potential overuse of cesarean in LMIC,^31^ any quality improvement work that introduces Doppler, particularly continuous monitoring, should also monitor potential overuse of this intervention.

### 4.2 Doppler to improve measurement of facility perinatal mortality

The WHO has called for a metric for perinatal mortality occurring after admission to a health facility that can be used to monitor quality of intrapartum care.^11^, ^32^ In two studies in five countries, Doppler was used to detect FHR among women on admission, allowing for verification of whether fetal deaths occurred before or after facility admission. This information is useful to calculate an indicator of perinatal mortality that occurs in a health facility (i.e., the mother was admitted to the facility with a documented FHR and was discharged with a stillborn or deceased newborn). It can be presumed that many of these cases represent poor quality of care.

Both of the studies concluded that such a facility perinatal mortality indicator is a feasible and useful measurement^21^, ^22^; one study also noted the feasibility of integrating the indicator into the national health information system.^22^ Despite the small number of studies, the findings support increased use of Doppler to accurately measure preventable perinatal death (intrapartum stillbirth and early neonatal death) occurring after admission to labor and delivery services in LMIC health facilities. Further studies might address the feasibility of integrating the indicator into health information management systems, provider acceptance of the indicator, costs associated with scaling up Doppler use, and national policy‐makers’ understanding of the need for the indicator.

### 4.3 Healthcare provider and maternal preference for Doppler as a means of FHR monitoring

The WHO considers maternal and healthcare provider preferences to be key elements for a positive childbirth experience,^9^ in addition to the importance of the woman having informed choices regarding interventions in labor.^27^ A strong maternal or healthcare provider preference for Doppler over Pinard may be sufficient to justify integrating the device into LMIC intrapartum care protocols. Three studies addressed healthcare provider and maternal preference for Doppler as compared with other devices for monitoring FHR. All three had substantial limitations regarding generalizability that restricts their utility in drawing programmatic or policy conclusions. The current evidence on maternal and provider preferences should be bolstered with studies that have greater generalizability and include the perspectives of women who experienced deliveries with fetal distress.

### 4.4 Limitations

The review has some limitations. First, the findings rely on the quality of included studies. All studies that examined adverse perinatal outcomes were designed with perinatal outcomes as a secondary outcome measure, and hence had relatively low power to detect these differences. Second, two studies indicated that, although FHR monitoring protocols were properly followed due to study oversight, there were delays in proper case management, impacting perinatal death rates.^16^, ^17^ Third, the review did not include a meta‐analysis owing to dissimilarity of interventions and outcome measures among the studies. Last, none of the included studies addressed the feasibility of scaling‐up use of Doppler, which would require an assessment of infrastructure‐related needs such as power, ultrasound gel, and maintenance, and which will ultimately be an important consideration in Doppler scale‐up In LMIC.

## 5 CONCLUSIONS

On the basis of the reviewed studies, it is reasonable to conclude that Doppler may be a better diagnostic tool than Pinard fetoscope for monitoring FHR in the LMIC facility setting. In all but a few cases, the studies that assessed interim measures of clinical management (i.e., cesarean delivery, intrauterine resuscitation, and time from detection of abnormal FHR to delivery) showed that these interventions were the same in the Doppler group as in the other groups, indicating a gap in clinical management after the detection of FHR abnormalities. Further research and programming should link intrapartum FHR monitoring using Doppler to improved clinical decision‐making, case management, and referral protocols in cases where an abnormal FHR is detected.

## AUTHOR CONTRIBUTIONS

MP and BK screened the search results and selected studies for inclusion. SW resolved conflicts in the review and screening process. MP and BK extracted data from studies and BK assessed risk of bias. LF and SW provided input to the content of the tables. JR, LF, SC and HK contributed substantially to interpretation of the extracted data and shaped the recommendations and implications of the findings. All authors read, revised and approved the final manuscript.

## CONFLICTS OF INTEREST

The authors have no conflicts of interest.
